# Adenine base editing to treat progeria syndrome and extend the lifespan

**DOI:** 10.20517/jca.2021.10

**Published:** 2021-06-17

**Authors:** Kiran Musunuru

**Affiliations:** Division of Cardiology and Cardiovascular Institute, Department of Medicine, Perelman School of Medicine at the University of Pennsylvania, Philadelphia, PA 19104, USA.

## Abstract

Hutchinson-Gilford progeria syndrome (HGPS) is an exceedingly rare and hitherto incurable and fatal disease marked by accelerated aging simultaneously affecting a number of organs. Most cases of HGPS are caused by a single copy of a specific single-nucleotide mutation, c.C1824T, in the *LMNA* (lamin A) gene. Different mutations in *LMNA* are responsible for a variety of disorders affecting a variety of organs, including dilated cardiomyopathy, familial partial lipodystrophy, Emery-Dreifuss muscular dystrophy, limb girdle muscular dystrophy, Charcot-Marie-Tooth disease, and restrictive dermopathy. The unique pathophysiology of HGPS arises from the distinctive nature of the c.C1824T mutation; despite being a synonymous mutation that does not directly change an amino acid in the lamin A protein, it nonetheless exerts a profound effect on the protein by creating a cryptic splice site that causes incorrect splicing of the *LMNA* mRNA transcript, resulting in production of a truncated form of lamin A termed progerin, which is constitutively farnesylated. The farnesylated protein inappropriately accumulates in cells and causes dysregulation of the nuclear lamina - a structure in which the normal lamin A protein is a key component - that results in cellular dysfunction, senescence, and death. Vascular smooth muscle cells (VSMCs) represent one of the cell types particularly affected by progerin, and cardiovascular complications are the typical cause of death of HGPS patients in their youth.

Various strategies to mitigate the effects of the *LMNA* c.C1824T mutation have been explored. Inhibitors of protein farnesylation have demonstrated efficacy in clinical trials with HGPS patients, with improvement of some aspects of the disease and even a modest increase in length of survival^[1]^. Despite being a promising approach that has received approval from the United States Food and Drug Administration for the treatment of HGPS, inhibition of farnesylation is limited in its efficacy and therefore insufficient on its own; furthermore, it produces substantial gastrointestinal side effects. Antisense oligonucleotides intended to specifically reduce the progerin-expressing, aberrant splice form of *LMNA* have proven effective in a mouse model of HGPS, substantially increasing the lifespans of the mice (by 62% in one study)^[2,3]^. While more specific and, at least in preclinical models, more effective than inhibition of farnesylation, the antisense oligonucleotides have the disadvantage that they require chronic, frequent injections in order to maintain their therapeutic effects.

Genome editing, in contrast, has the potential to provide “one-and-done” treatments that modify the *LMNA* gene at the DNA level and thus permanently suppress progerin production. Two proof-of-concept studies used the CRISPR-Cas9 genome-editing system to target near or at the site of the *LMNA* c.C1824T mutation, with the goal of introducing double-strand breaks that are incorrectly repaired via non-homologous end-joining, resulting in insertion or deletion (indel) mutations that disrupt the progerin protein^[4,5]^. (Lamin A is also disrupted, but a shorter isoform expressed from the same gene, lamin C, is still intact and can compensate for loss of lamin A in mice.) The studies used adeno-associated viral (AAV) vectors to deliver Cas9 and guide RNAs - the former providing the “cutting” function and the latter providing the “GPS” function directing Cas9 to the desired genomic location - into various organs in the bodies of mice, which resulted in the desired editing. Besides producing beneficial changes at the cellular level, the single AAV treatments extended the lifespans of the mice, ~25% in each study. Despite the successes, the consequences of reduction of lamin A along with progerin in human cells remain unclear, and the stochastic nature of the indel mutations raises the possibility of unintended consequences of the genome editing.

In principle, a newer form of genome editing called base editing can overcome the limitations of standard CRISPR-Cas9 genome editing^[6]^. Base editing uses a catalytically impaired “nickase” form of CRISPR-Cas9 - capable of breaking only one strand of DNA at the target location, rather than a double-strand break - fused to an enzymatic domain that can chemically modify DNA bases [Figure 1]. The two major types of base editing reported to date use different enzymatic domains. In cytosine base editing, the nickase CRISPR-Cas9 directs a fused deoxycytidine deaminase domain, adapted from any of a large number of naturally occurring DNA deaminases, to a specific site in the genome. Within a short window of single-strand DNA sequence made accessible to the deaminase domain by CRISPR-Cas9 unwinding the DNA double helix at the target location, any cytosine base can be modified to uracil. Nicking of the opposite DNA strand and the consequent DNA repair process results in replacement of the guanine base that was opposite the edited cytosine with an adenine (complementing the uracil). Ultimately, the uracil - which is recognized by the cell as not naturally occurring in DNA - is replaced with thymine, finalizing a C-G base pair conversion to a T-A base pair (C-to-T edit). By an analogous mechanism, in adenine base editing, a novel deoxyadenosine deaminase domain created by molecular evolution (of a naturally occurring domain that only acts upon RNA) converts any adenosine within the editing window into inosine (I) and, ultimately, guanosine (A-to-G edit).

If a C-to-T or A-to-G edit can directly correct a disease-causing mutation, a base editor can potentially make that edit in a precise fashion that avoids the stochastic indel mutagenesis of standard genome editing. Fortuitously, the prevalent mutation underlying HGPS, *LMNA* c.C1824T, can be directly corrected by an A-to-G edit on the antisense strand (editing of the base in the position opposite the c.C1824T base). Unlike any other therapeutic approach entertained to date, adenine base editing can fully reverse the primary genetic defect in HGPS, eliminating the production of the pathogenic progerin protein while preserving the normal lamin A protein.

Dr. Koblan *et al.*^[7]^ recently provided a compelling proof-of-concept demonstration of adenine base editing to ameliorate progeria phenotypes in a mouse model of HGPS. The mouse model in question was homozygous for a human *LMNA* transgene harboring the c.C1824T mutation, resulting in VSMC defects, hair loss, lack of subcutaneous fat, musculoskeletal abnormalities, and shortened lifespan, phenocopying some characteristics of HGPS patients. The investigators began by testing adenine base editors in fibroblast cell lines derived from HGPS patients. They found that a combination of an adenine base editor called ABEmax-VRQR and a particular guide RNA, when delivered together into the cells via a lentiviral vector, could achieve 80%-90% correction of the mutation, with very low levels of undesired edits at the target site (1%-2%). There were concomitant 6-15-fold reductions in progerin levels in the treated cells. The investigators performed a variety of analyses to assess for any off-target editing by the adenine base editor - that is, unintended edits in the DNA genome at sites other than the desired target site in *LMNA*, as well as unintended edits in the RNA transcriptome - and were not able to detect any such editing.

The investigators then used AAV vectors to deliver ABEmax-VRQR and the guide RNA into a variety of tissues in the HGPS mouse model. Because the gene encoding the adenine base editor was too large to fit in a single AAV vector, the authors split the editor into two parts that were designed to spontaneously assemble into a single functional protein via intein-mediated protein splicing, delivering the base editing system via two AAV vectors. In testing various injection routes and postnatal injection times, they were able to achieve up to ~60% mean editing in liver, ~30% mean editing in heart and quadriceps, and ~20% mean editing in aorta and bone. They then performed a long-term study in which they could assess for phenotypic amelioration in treated *vs.* control (saline-injected) HGPS mice. In heart and aorta, they observed mean reductions in progerin levels of 86% and 49%, respectively, six months after treatment. Whereas control HGPS mice displayed a loss of VSMCs in aortic vessel walls and periadventitial thickening, the treated HGPS mice had essentially complete rescue of these phenotypes, resembling wild-type mice. Perhaps the most dramatic observation was that the treated HGPS mice lived 2.4 times as long as the control HGPS mice - an extension of lifespan surpassing other treatment modalities, speaking to the utility of direct correction of the primary genetic defect in HGPS.

The single red flag was that the majority of the long-lived treated HGPS mice, upon expiration, were found to have liver tumors. Further investigation suggested that AAV integration into genomic regions where AAV integration has been previously associated with liver tumors in mice may have been responsible for these tumors, rather than the effects of the adenine base editing. The recent demonstration of delivery of short-lived adenine base editor mRNA and guide RNA via lipid nanoparticles into liver in non-human primates, resulting in durable, near-saturation editing of the *PCSK9* gene^[8]^, suggests that non-viral approaches may ultimately prove to be more useful than AAV vectors in the treatment of HGPS patients, pending the development of lipid nanoparticles that can target the vasculature and other non-liver organs. Furthermore, the recent creation of a new type of genome editing, prime editing, that allows for any possible DNA base substitution as well as precise sequence insertions and deletions^[6]^ may prove even more advantageous than base editing in correcting the HGPS mutation and other laminopathy mutations. Nonetheless, the work of Liu, Brown, Collins, and colleagues represents an important step forward - not just with respect to the treatment of HGPS patients, but for the aging and genome editing fields as well.

## Figures and Tables

**Figure 1.**
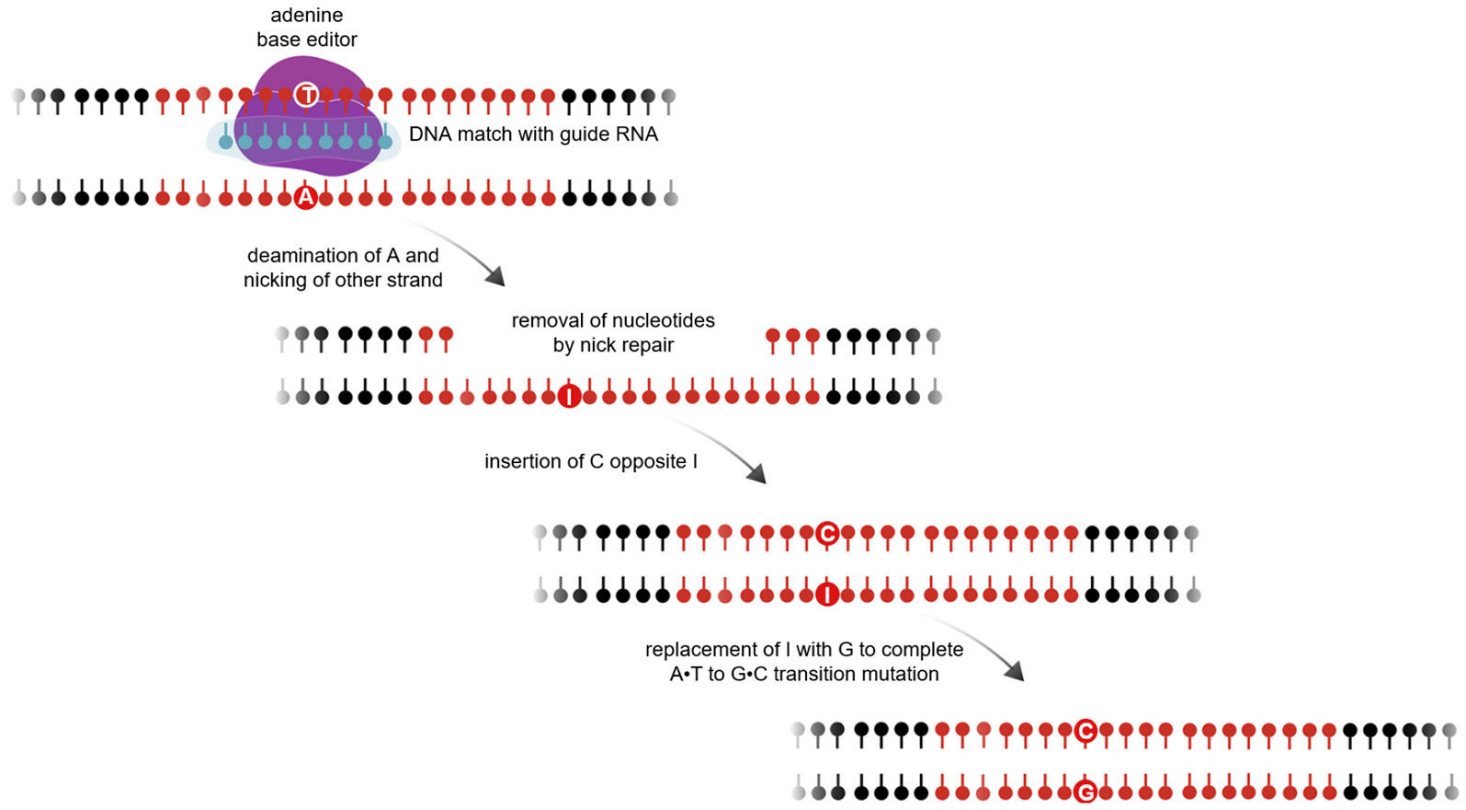
Schematic of adenine base editing. First published in Musunuru *et al.*^[8]^, *Nature* 2021;593:429-34, by Springer Nature.
